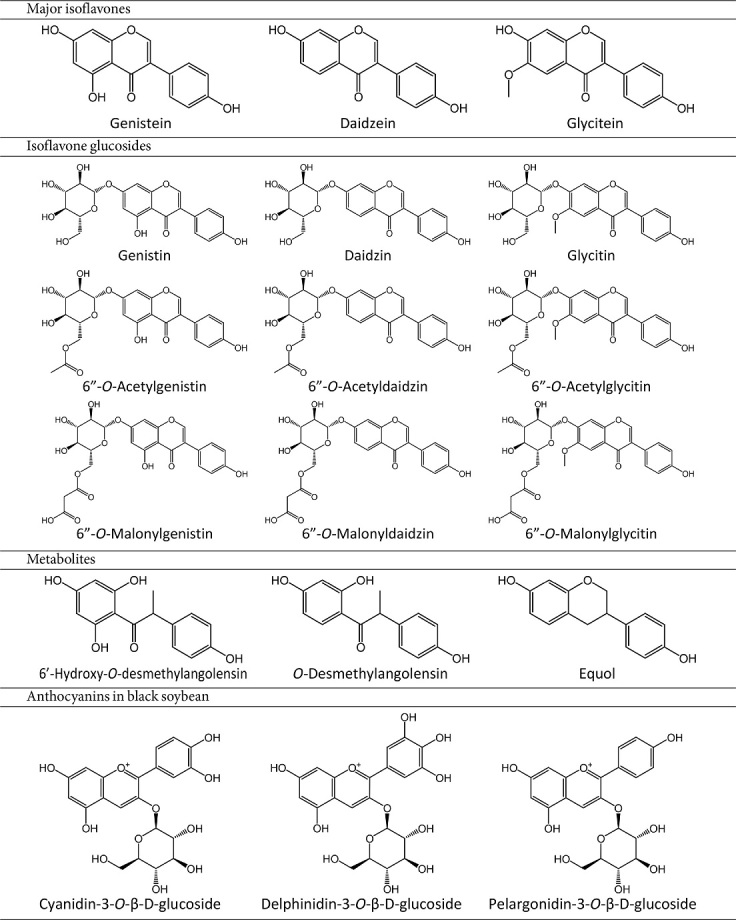# Benefits of Soybean in the Era of Precision Medicine: A Review of Clinical Evidence

**DOI:** 10.4014/jmb.2308.08016

**Published:** 2023-08-28

**Authors:** Jung Hyun Kang, Zigang Dong, Seung Ho Shin

**Affiliations:** 1 Department of Food and Nutrition, Gyeongsang National University, Jinju 52828, Republic of Korea; 2 Department of Pathophysiology, School of Basic Medical Sciences, Zhengzhou University, Zhengzhou 450001, Henan, P.R. China; 3 China-US (Henan) Hormel Cancer Institute, No.127, Dongming Road, Jinshui District, Zhengzhou 450008, Henan, P.R. China; 4 Department of Bio & Medical Bigdata (BK4 Program), Gyeongsang National University, Jinju 52828, Republic of Korea

**Keywords:** Soybean, soy isoflavone, meta-analysis, precision medicine

## Abstract

Soybean (*Glycine max*) is an important ingredient of cuisines worldwide. While there is a wealth of evidence that soybean could be a good source of macronutrients and phytochemicals with health-promoting effects, concerns regarding adverse effects have been raised. In this work, we reviewed the current clinical evidence focusing on the benefits and risks of soybean ingredients. In breast, prostate, colorectal, ovarian, and lung cancer, epidemiological studies showed an inverse association between soybean food intake and cancer risks. Soybean intake was inversely correlated with risks of type 2 diabetes mellitus (T2DM), and soy isoflavones ameliorated osteoporosis and hot flashes. Notably, soybean was one of the dietary protein sources that may reduce the risk of breast cancer and T2DM. However, soybean had adverse effects on certain types of drug treatment and caused allergies. In sum, this work provides useful considerations for planning clinical soybean research and selecting dietary protein sources for human health.

Soybean has been an important ingredient of cuisines in many countries for centuries [1-3]. Asian countries have consumed soybean in many ways, including fermented soybean [4-10], tofu [11], and soymilk [12]. The availability of soybean-based products in the U.S. and other Western countries has increased over the past few decades [13, 14]. Given the dietary recommendation encouraging plant-based meals [15-17], soybean has become a good source of high-quality proteins and phytochemicals.

Regarding the effects of soybean, ambiguous results and limitations exist. In coronary heart disease, for example, the Food and Drug Administration (FDA) is considering revoking soy protein’s heart health claim due to a perceived lack of consistent effects [18, 19]. In some cases, the beneficial effects of soybean in vitro or in vivo were not shown in the clinical studies. It is partly because most animals metabolize soy isoflavones differently than human [20]. Moreover, specific bioactive components of soy and their detailed molecular mechanism in human are poorly understood. The correlation between soybean intake and the genetic features of participants or patients is not well-known. Therefore, a comprehensive review of soybean compiling clinical data that has been reported thus far to utilize the food source in human better.

Major clinical symptoms and diseases worth discussing include cancer, diabetes, and postmenopausal symptoms such as osteoporosis and hot flashes. Cancer is a disease with the highest mortality rate worldwide, along with cardiovascular disease [21]. Prostate cancer and breast cancer rank first in incidence and second in mortality in men and women worldwide [22]. In addition, lung and colorectal cancer incidence and mortality rank first and third, respectively [22]. While diabetes has increased significantly in prevalence in all parts of the world in recent decades [23], its threat to health has been underestimated [24]. However, complications of diabetes, such as renal failure, cerebrovascular disease, and coronary artery disease, are one of the most critical public health problems of the 21st century, increasing patient mortality [24, 25]. Osteoporosis and hot flashes are frequent in postmenopausal women and are accelerated by decreased estrogen secretion [26]. In an aging society, the health of postmenopausal women is emerging as a social problem and high-quality protein intake is required to address the issue [27].

Here, we summarize the major bioactive components of soybean and their consumption in the human body. Epidemiological studies of soybean and its bioactive components are reviewed to show its association with major types of cancer and other disorders, including type 2 diabetes mellitus (T2DM), osteoporosis, and hot flashes. Meta-analyses on randomized controlled trials and other intervention studies show the effect of soybean consumption on diseases. We present a balanced view of soybean by providing its beneficial and adverse effects and suggest future directions of soybean research in precision medicine.

## Major Soybean Components and Human Consumption

Soybean reportedly comprises 35–40% protein, 20% fat, 9% dietary fiber, and 8.5% moisture [28], but their compositions vary with their variety, planting location, and climate [29]. Major soy components with biological activities include peptides [30-36] and isoflavones [37-39].

### Protein (Peptide)

Soybean contains higher protein than other vegetable sources and has become an alternative to animal protein [40-42]. β-Conglycinin and glycinin are two major proteins in soybean, comprising 80-90% of soy protein [28]. Isolated soy protein or its concentrate shows good digestibility in the body [28]. Generally, peptides are produced by digestion of soybean proteins, which are 2-20 amino acids long. Peptides are absorbed into the small intestinal epithelial cells and then exported into the blood circulation [43].

Bioactive peptides remain inactive in their parent protein sequences [44] but become bioactive when released by fermentation, enzymatic processing, or gastrointestinal digestion [28]. Fermentation refers to producing different soy-based products from soybeans using bacteria such as *Bacillus subtilis* and *Aspergillus*, and it is one of the standard methods of metabolizing soy proteins outside of the human body [45, 46]. The sensory and functional properties of soy are increased by fermentation due to the enzymatic breakdown of proteins into peptides [45].

Asian countries consume much more soy protein than Western countries. The average daily soy protein intakes in different countries are as follows: 6-11.3 g in Japan [47], 2.5-10.3 g in China [40, 48], 3.8-8.5 g in Korea [49, 50], 4.2-6.6 g in Hong Kong [51], and 1 g or less in the United States [51-53].

### Phytochemical

The most abundant type of phytochemical in soybean is isoflavone [54]. Soy isoflavones are bioactive small molecules with non-steroidal and phenolic nature [55]. They are often called phytoestrogens because of their structural similarity to 17-β-estradiol, which can bind to estrogen receptor (ER) [56, 57]. Major isoflavones biosynthesized in soybean are glucosides: genistin, daidzin, and glycitin (Table 1). Besides the glucosides, they also exist as acetyl glucoside or malonyl glucoside forms [58]. After the metabolism process through digestion or fermentation by β-glucosidases, they are converted to bioactive aglycones [39]. Genistein, daidzein, and glycitein, the most abundant aglycones in edible forms, are found approximately at 58:37:5 ratio if their derivatives are included [59]. Depending on the color of the soybean, additional phytochemicals can be detected. The pigmentation of black soybean, for example, is due to high amounts of unique anthocyanins, including cyanidin-3-*O*-β-glucoside, delphinidin-3-*O*-β-glucoside, and pelagonidin-3-*O*-β-glucoside (Table 1) [60].

Gut microbiome and Fermentation are two central mechanisms of soy isoflavone metabolism. Genistein is hydrolyzed to dihydrogenistein and then converted to 6’-hydroxy-*O*-desmethylangolensin in the human body (Table 1) [61]. Similarly, daidzein is hydrolyzed and converted to O-desmethylangolensin and equol in the human intestine or bacteria [62]. Genistein, daidzein, and equol are the most abundant isoflavones detected in blood and urine [63]. They persist in the plasma for 24 h with an average half-life of 6-8 h [64].

**Fig. 1:**
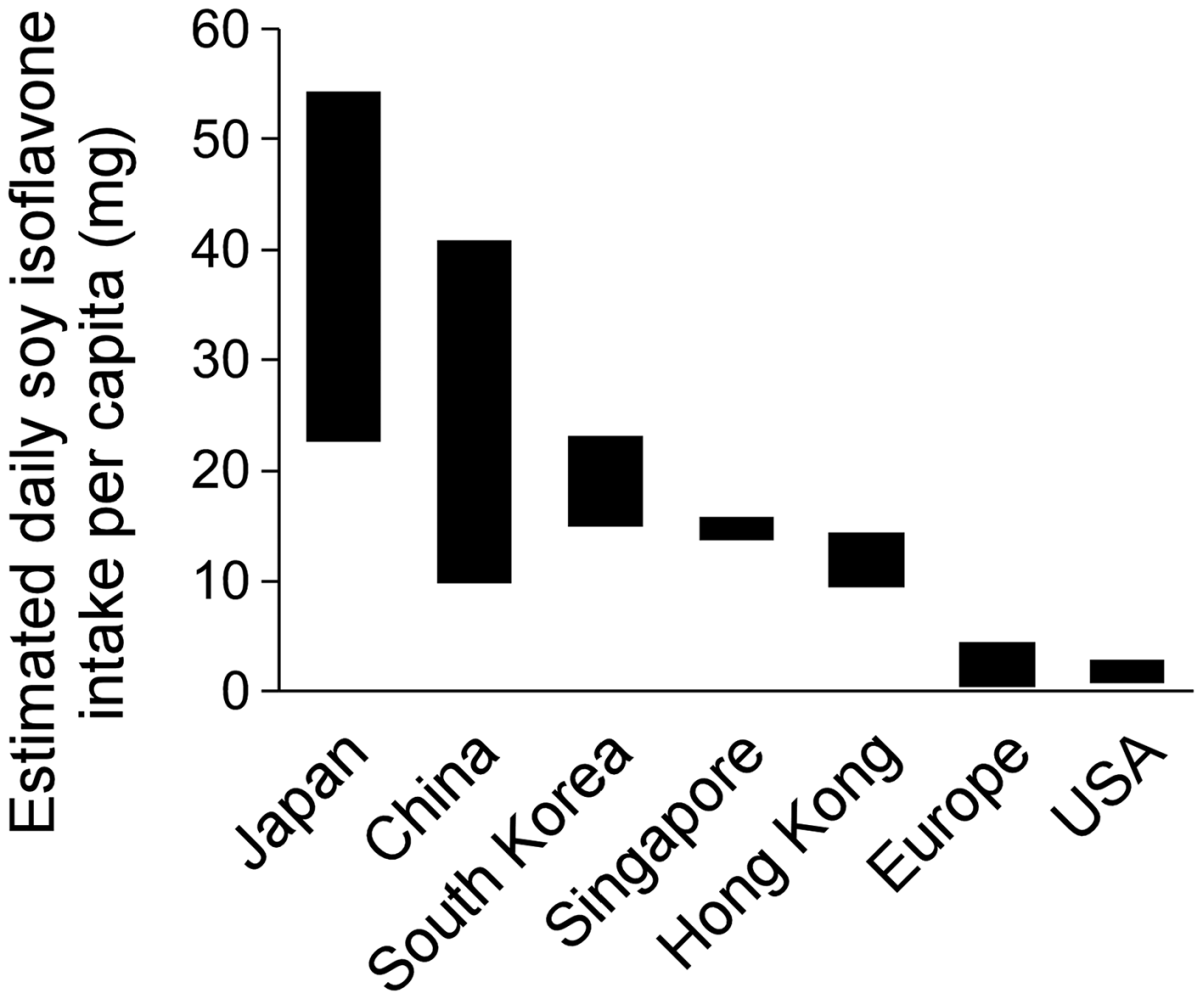
Estimated ranges of daily soy isoflavone intake per person across different countries.

The daily isoflavone intake is a good indicator correlated with soybean consumption. The highest isoflavone intake is in East and South Asian countries (Fig. 1). Soy isoflavone intake in Japan was reported to be 22.6-54.3 mg/day [47, 52, 65-69]. China also showed a wide range of isoflavone consumption levels from 9.8 to 40.9 mg/day [48, 70-73]. In South Korea, soy isoflavone intake was estimated to be between 14.9 and 23.1 mg/day [50, 74, 75]. The soy isoflavone intakes in Singapore and Hong Kong were similar to the lowest intake quartile of China, showing ranges of 13.7-15.9 mg/day [47, 76] and 9.5-14.5 mg/day [51], respectively. In sharp contrast, daily isoflavone consumption in Europe and the United States was only 0.37-4.5 mg/day [40] and 0.49-1 mg/day [77, 78].

### Fat

Fat or lipid contents of soybean are comprised of polyunsaturated fatty acids (PUFA, 46-63%), monounsaturated fatty acids (20-41%), and saturated fatty acids (10-15%) [79]. Among PUFA, linoleic acid (omega-6 fatty acid) and α-linolenic acid (omega-3 fatty acid) comprise 88.2% and 11.8%, respectively. Because soybean oil is the most widely used edible oil in the U.S., it accounts for over 40% of omega-3 and omega-6 fatty acid intake [80].

## Beneficial Effects of Soybean

**Fig. 2:**
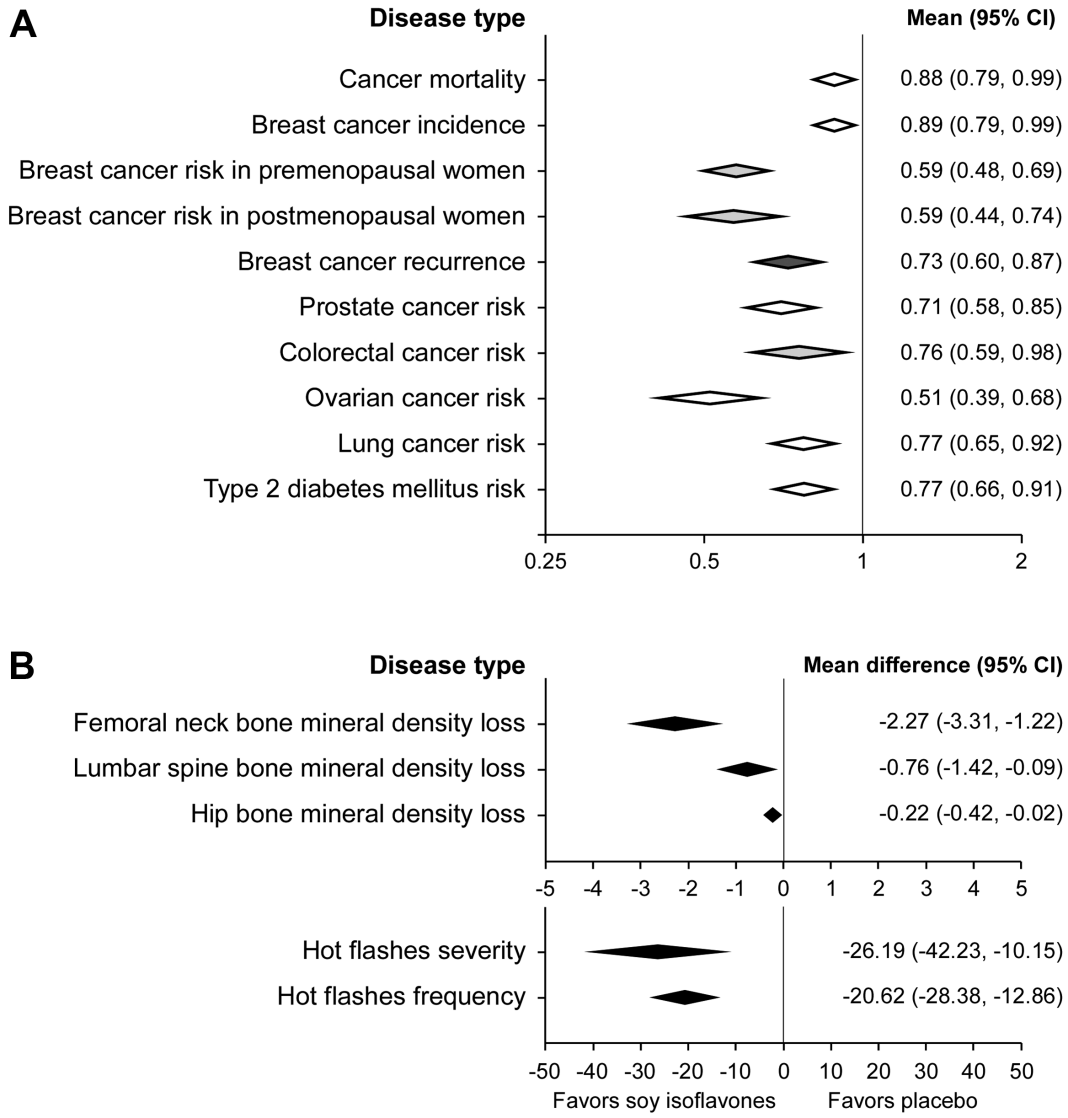
Forest plot summary of clinical evidence related to soybean intake. (**A**) Meta-analyses of soybean and soy isoflavone intake against different diseases. The color of diamonds depicts different ratios/risks; white: RR (relative risk), light grey: OR (odds ratio), and dark grey: HR (hazard ratio). (**B**) Meta-analyses of randomized controlled trials of soy isoflavones. Negative values of mean difference favor soy isoflavone intake.

Considerable research efforts have been made to find the benefits of soybean in cancer prevention and treatment. Soybean and soy product consumption was inversely associated with cancer deaths in general [81]. Nachvak *et al*. performed a meta-analysis of nine prospective studies with a total sample size of 165,228 participants and 9,804 cancer deaths and showed that soy/soy product consumption reduced relative risk (RR) on cancer mortality (pooled RR = 0.88, 95% confidence interval (CI) = 0.79-0.99; Fig. 2A) [81]. In particular, epidemiological evidence shows that soybean intake has inverse associations with multiple cancer types, including breast, prostate, lung, and colon [82].

### Breast Cancer

Phytoestrogen is a naturally-occurring compound found in plants such as soybean and can act as a competitive agonist of ER due to their chemical similarity with estrogen [82]. The involvement of ER [83] in breast cancer, which accounts for 31.4% of all new cancer diagnoses among women in 2023 [22], led researchers to investigate the association between soybean intake and breast cancer risks. Many meta-analyses have shown that soybean or isoflavone intake was inversely associated with the risk of breast cancer incidence [84-90]. A meta-analysis of 14 breast cancer incidence studies showed that soy isoflavone consumption was inversely correlated with the risk of breast cancer incidence (RR = 0.89, 95% CI = 0.79–0.99; Fig. 2A) [84]. Another meta-analysis of 35 studies revealed that the protective effect of soy isoflavones was seen in both premenopausal women (odds ratio (OR) = 0.59, 95% CI = 0.48–0.69) and postmenopausal women (OR = 0.59, 95% CI = 0.44–0.74; Fig. 2A) [85]. A recent case-control study of breast cancer risk factors in 7,663 women in Malaysia also showed that a higher soymilk and soy product intake was associated with a lower risk of breast cancer [91]. The group of women who take one or more glasses of soy milk per week had an OR of 0.25 (95% CI = 0.18-0.33). The women taking soy products once a week or more were also associated with reduced breast cancer risk (OR = 0.40, 95% CI = 0.33-0.48) [91].

Consumption of soy food and soy isoflavone is also associated with a lower risk of breast cancer recurrence among breast cancer patients [92]. Qiu and Jiang performed a meta-analysis [92] to find a correlation between breast cancer recurrence and soy/soy isoflavone consumption at pre-diagnosis [93, 94]. Soy and isoflavone intake was associated with reduced breast cancer recurrence risk (hazard ratio (HR) = 0.73, 95% CI = 0.60-0.87; Fig. 2A)[92]. Chi *et al*. conducted another meta-analysis to find a correlation between the recurrence and soy food consumption post-diagnosis [95]. Soy food intake was associated with a reduced risk of breast cancer recurrence (HR = 0.79, 95% CI = 0.72-0.87) [95].

The associations between soybean consumption and different subtypes of breast cancer patients have been revealed, accelerating the momentum of precision oncology. Baglia *et al*. conducted a prospective study of 70,578 Chinese women with a median follow-up of 13.2 years and 1,034 breast cancer incidences [96]. Soy intake was associated with reduced risk of ER+/progesterone receptor (PR)+ breast cancer in postmenopausal women (HR = 0.72, 95% CI: 0.53–0.96) and reduced risk of ER-/PR- breast cancer in premenopausal women (HR = 0.46, 95% CI: 0.22–0.97) [96]. Moreover, soy food consumption was also associated with a lower risk of recurrence in ER-negative (highest vs. lowest quintile: HR = 0.64, 95% CI = 0.44-0.94) and ER+/PR+ (highest vs. lowest: HR = 0.65, 95% CI = 0.49-0.86) breast cancer patients [95]. For postmenopausal patients, soy food intake was also correlated with reduced recurrence (highest vs. lowest: HR: 0.67, 95% CI: 0.56-0.80) [95]. In summary, soy food and isoflavone consumption is associated with reduced breast cancer incidence and recurrence, and analyses stratified by hormone receptor and menopausal status allow researchers to understand soybean’s effect on breast cancer better.

Notably, soybean is one of the protein sources that may reduce the risk of breast cancer among ten different dietary protein sources [97]. A meta-analysis study investigated the association between breast cancer risk and different dietary protein sources: fresh red meat, processed meat, poultry, fish, egg, soy food, nuts, whole milk, skim milk, and yogurt [97]. The relative risk (RR) for the highest versus lowest soy food intake was 0.92 (95% CI = 0.84-1.00). A similar conclusion was found in the dose-response association for soy food (RR = 0.91; 95% CI = 0.84-1.00), indicating that higher soy food intake may reduce the risk of breast cancer. Skim milk was the other protein source with a beneficial effect showing RR = 0.96 (CI = 0.92-1.00) for the dose-response association. On the other hand, total red meat (RR = 1.07; 95% CI = 1.01-1.14), fresh red meat (RR = 1.13; 95% CI = 1.01–1.26), and processed meat (RR = 1.09; 95% CI = 1.02-1.17) were potential risk factors for breast cancer in the dose-response association analyses [97]. In conclusion, soybean is a desirable choice when selecting protein sources with the potential benefit of reducing breast cancer risk.

The beneficial effect of soybean against breast cancer risk was evident in countries with high-soy-consuming populations [98, 99]. A meta-analysis of eight studies conducted in Asia and Asian Americans and 11 studies conducted in Western populations showed a significant decreasing risk trend with increasing soy food intake in Asians [99]. There was an approximately 16% risk reduction per 10 mg of soy isoflavones intake per day in Asian populations. The breast cancer risk was intermediate (OR = 0.88, 95% CI = 0.78–0.98) for those who consume 10 mg or more of isoflavones/day and the lowest (OR = 0.71, 95% CI = 0.60–0.85) for those who consume 20 mg or more of isoflavones/day, compared to the lowest level of soy food intake (less than 5 mg of isoflavones/day) [99]. In contrast, there was no correlation between soy intake and breast cancer risk in Western populations whose average highest and lowest soy isoflavone intake levels (0.8 and 0.15 mg/day, respectively) are far lower than those of Asian countries.

### Prostate Cancer

Prostate cancer is the most commonly diagnosed cancer among men, comprising 28.5% of new cancer cases in 2023 [22]. The association between soybean and prostate cancer has been investigated based on epidemiological evidence that the incidence and mortality rates of prostate cancer in Asian countries with high soybean consumption are lower than in Western countries [100]. A meta-analysis including 30 research articles, 266,699 participants, and 21,612 prostate cancer cases revealed correlations between soybean and prostate cancer risks [101]. The pooled RR associated with prostate cancer for total soy food intake was 0.71 (95% CI = 0.58-0.85; Fig. 2A). Subgroup analyses showed that dietary genistein was associated with reduced prostate cancer risk (pooled RR = 0.90, 95% CI = 0.84-0.97). Dietary daidzein was also significantly correlated with the reduced risk (pooled RR = 0.84, 95% CI = 0.73-0.97). When soy food was stratified by unfermented and fermented ones, unfermented soy food was associated with reduced prostate cancer risk (pooled RR = 0.66, 95% CI = 0.52-0.83), but fermented soy food had no significance (pooled RR = 0.86, 95% CI = 0.66–1.13, *p*-value = 0.281). In summary, total soy, genistein, daidzein, and unfermented soy food comsumptions are correlated with prostate cancer risk reduction.

Concerning cancer intervention, however, there is limited evidence of whether soybean consumption is correlated with suppression of prostate cancer progression or recurrence. Short-term consumption of soybean caused a decrease in prostate-specific antigen (PSA), a biomarker of benign prostatic hyperplasia or prostate cancer [102], or a decline in the increasing rate of PSA in a small subset of patients with recurrent prostate cancer [103]. In contrast, long-term soybean consumption did not affect prostate cancer progression [104] nor recurrence [105]. 40 g of daily soy consumption and other micronutrient supplements (vitamin E and selenium) showed no difference compared to placebo (HR = 1.03, 95% CI = 0.67-1.60) [104]. In the other randomized, double-blind trial, Bosland *et al*. used soy protein supplements with 177 men at high risk of prostate cancer after radical prostatectomy [105]. 27.2% (22/81 participants) of the intervention group and 29.5% (23/78 participants) of the placebo group developed recurrence within two years of the trial, showing that daily soy protein consumption did not reduce prostate cancer recurrence.

### Other Cancer Types

Unlike breast and prostate cancer, clinical data related to other cancer types are limited. Soybean or isoflavone consumption is also associated with the reduction of colorectal, ovarian, and lung cancer risks.

In colorectal cancer, the association with reduction of the cancer risk is more evident for isoflavones than total soybean. A small cohort study conducted in Korea and Vietnam, countries with high soybean consumption, showed that genistein and daidzein are correlated with a significantly decreased risk of colorectal cancer (OR = 0.46, 95% CI = 0.30-0.69 for genistein; OR = 0.54, 95% CI = 0.36-0.82 for daidzein) [106]. A meta-analysis by Tse and Eslick showed that total soybean intake is only associated with a slight decrease in colorectal cancer risk [107]. The adjusted OR of soybean consumption for colorectal cancer was only 0.92 (95% CI = 0.87-0.97). Subgroup analysis for soy isoflavone showed a more significant risk reduction against colorectal cancer (OR = 0.76, 95% CI = 0.59-0.98; Fig. 2A). The other meta-analysis using total soybean showed that soybean was associated with a 21%reduction in colorectal cancer risk in women but not men [108].

In ovarian cancer, soybean and soy isoflavone showed their possibility to reduce ovarian cancer risk. Isoflavone was associated with decreased ovarian cancer risk (RR = 0.67, 95% CI = 0.50–0.92) in a meta-analysis by Hua *et al*. in which 12 studies were included [109]. In the other meta-analysis conducted by Qu *et al*., soy food intake (RR = 0.51, 95% CI = 0.39-0.68) and soy isoflavone (RR = 0.63, 95% CI = 0.46-0.86) showed their association with reduced ovarian cancer incidence (Fig. 2A) [110]. However, the authors noted that larger and well-designed observational studies in ovarian cancer are needed to characterize the correlations fully [109, 110].

In lung cancer, the association between lung cancer risk decrease and soy intake is reported, but the effect of soy protein or isoflavone is unclear. A meta-analysis by Yang *et al*. showed a significantly inverse association between soy intake and lung cancer (RR = 0.77, 95% CI = 0.65-0.92; Fig. 2A) [111]. Another meta-analysis investigating the role of daily soy protein consumption against lung cancer found only a marginally inverse association (OR = 0.98, 95% CI = 0.96-1.00) [112]. In summary, soy intake showed promising inverse correlations with risk reduction of colorectal, ovarian, and lung cancer; however, more case-control and prospective cohort studies are needed to find a more evident association.

### Type 2 Diabetes Mellitus (T2DM)

According to the Centers for Disease Control and Prevention, about 37.3 million people accounting for 11.3%of the U.S. population, suffer from diabetes mellitus, and 90 to 95% have T2DM [113]. Soybean intake is associated with a lower risk of T2DM [114, 115]. Li *et al*. conducted a meta-analysis of eight observational studies on soy products and T2DM [114]. Inverse associations between reduced T2DM risk were observed with total soy intake (RR = 0.77, 95% CI = 0.66-0.91; Fig. 2A) and soy protein/isoflavone (RR = 0.88, 95% CI = 0.80-0.97). In the subgroup analysis, soybean consumption was associated with reduced risk in women (RR = 0.65, 95% CI = 0.49–0.87) but not in men (RR = 0.82, 95% CI = 0.58-1.16, not significant), which implies possible involvement of estrogen receptor-mediated activities.

Notably, soy is a good protein source significantly associated with reduced risk of T2DM in women. Tian *et al*. investigated the relationship between dietary protein intake and the risk of T2DM with 483,174 participants and 52,637 T2DM cases from eleven cohort studies [116]. They found that plant protein was weakly associated with reduced T2DM risk in women (RR = 0.92, 95% CI = 0.86–0.98) but not in men (RR = 0.98, 95% CI = 0.72–1.34, not significant), whereas animal protein was associated with increased risk of T2DM in both genders (RR = 1.23, 95%CI = 1.09-1.38 in men; RR = 1.11, 95% CI = 1.03-1.19 in women). Among the plant proteins, soybean was associated with a lower risk of T2DM in women (RR = 0.74, 95% CI = 0.59-0.93). This result indicates that the choice of dietary protein source might affect T2DM risk and that soybean can be considered for preventing T2DM in women especially.

Regarding the intervention of T2DM, however, the effect of soybean is minimal. Liu *et al*. [117] and Yang *et al*.[118] conducted systematic reviews and meta-analyses of soybean consumption on T2DM markers. The intake of soy products had no significant effect on fasting glucose level, insulin, and glycated hemoglobin, a marker of the average blood glucose level [117, 118]. Although soy consumption reduced serum levels of total cholesterol, triacylglycerol (TG), and low-density lipoprotein-cholesterol (LDL-C) and increased that of high-density-lipoprotein-cholesterol of T2DM patients, subgroup analysis showed that the reduction of TG and LDL-C levels was only temporary [118]. When followed up more than eight weeks, there was no significance between soy consumption and the reduction of TG and LDL-C levels. In summary, the intake of soy and soy products modulates serum lipid levels, but no robust effect was found on any endpoints in T2DM patients.

### Osteoporosis

Osteoporosis is a skeletal condition in which body bones lose their mass and show a highly porous structure with increased bone fragility and susceptibility to fracture [119]. Decreased bone mineral density (BMD) measured by X-ray absorptiometry is a gold standard for the diagnosis of osteoporosis [120], and bone formation markers such as osteoprotegerin and osteocalcin and bone resorption markers such as pyridinamine and C-telopeptide are commonly measured [121]. The osteoporosis risk in postmenopausal women increases due to a rapid decrease in estrogen concentration and increased bone remodeling. The BMD decreases in the lumbar spine and hip in postmenopausal women are at least 1% and 2.4%, respectively [122, 123]. Therefore, many women seek estrogen alternatives, such as soy isoflavones, to reduce osteoporosis risk.

Soy isoflavone consumption prevents bone resorption and mineral loss [124, 125]. A meta-analysis of randomized controlled trials by Akhlaghi *et al*. showed the effect of soy isoflavones on bone mineral density and osteoporosis markers in 5,313 participants [124]. Intake of soy isoflavones resulted in a significant increase in BMD mean difference (MD) in the femoral neck (MD = 2.27%, 95% CI = 1.22-3.31), lumbar spine (MD = 0.76%, 95% CI = 0.09-1.42), and hip (MD = 0.22%, 95% CI = 0.02-0.42; Fig. 2B). Subgroup analysis revealed that interventions longer than a year showed more significant effects: femoral neck (MD = 3.16, 95% CI = 1.57-4.75), lumbar spine (MD = 1.30, 95% CI = 0.43-2.16), and hip (MD = 0.38, 95% CI = 0.23-0.54). A bone formation marker, osteoprotegerin, was increased by 5.79 pg/ml (95% CI = 3.08-8.51) and two bone resorption markers, pyridinoline and C-telopeptide, were decreased by 5.13 nmol/mmol (95% CI =-7.76 to -2.50) and 0.08 ng/ml (95% CI = -0.16 to -0.00) by soy isoflavones, respectively. In summary, soy isoflavone reduces osteoporosis-related bone loss preferentially by a year-long intervention or more.

### Hot Flashes

Most postmenopausal women experience hot flashes, flushing, and night sweats, known as vasomotor symptoms. According to a 13-year-long observational study of the menopausal transition among 1,449 women, the average duration of hot flashes and night sweats was 7.4 years [126]. Soybean consumption has been suggested to avoid hormonal therapy, which can cause adverse effects for postmenopausal women for two reasons: the low prevalence of hot flashes among native Japanese women and the estrogen receptor as a molecular target of isoflavones [127].

Soy isoflavones reduce the frequency and severity of hot flashes in perimenopausal and postmenopausal women. Taku *et al*. performed a meta-analysis of 19 randomized controlled trials from 10 different countries to determine the efficacy of soy isoflavones on hot flashes [128]. Soy isoflavone consumption (mea*n* = 54 mg, duratio*n* = 6 weeks to 12 months) significantly reduced the severity of hot flashes by 26.19% (95% CI = -42.23 to -10.15; Fig. 2B). The frequency of hot flashes was also reduced by 20.62% (95% CI = -28.38 to -12.86) by soy isoflavone consumption (Fig. 2B). If more than 18.8 mg of genistein is included in isoflavone supplements, their effect on reducing hot flash frequency is more than twice compared to ones with a lower amount of genistein. Daily *et al*. also reported that equol, a microbiota-derived daidzein metabolite, decreases hot flashes in postmenopausal Women [129]. In summary, soy isoflavone consumption is not only associated with the low prevalence of hot flashes but also reduces the frequency and severity of the symptom in perimenopausal and postmenopausal women.

## Adverse Effects of Soybean

Although soybean provides many benefits, soybean ingredients possess risks for adverse interactions with breast cancer treatment. Like peanut allergy, soybean-based protein can be allergic to a group of people. The consumption of soybean above recommended doses can be harmful to the human body.

### Interference to Breast Cancer Treatment

Genistein was reported to negate the effectiveness of aromatase inhibitors against breast cancer in preclinical models [130]. Aromatase inhibitors such as fadrozole and letrozole block local estrogen production, which is essential for breast tumor growth and are prescribed to patients with estrogen-dependent breast cancer [131]. However, genistein increased breast cancer cell-associated aromatase expression and activity and thus induced the growth of estrogen-dependent MCF-7 breast cancer cell line [132]. When treated with fadrozole or letrozole, genistein negated the inhibitory activity of the aromatase inhibitors against estrogen-dependent breast cancer in vitro and in vivo [132, 133]. Therefore, postmenopausal women taking aromatase inhibitors for breast cancer treatment might need to be advised against taking soy products containing genistein [130].

### Allergic Disease

Allergic disease is a damaging immune response of the body caused by hypersensitivity of the immune system to typically harmless substances, including foods [134]. The term refers to a harmful biological response and differs from “allergy,” which includes body-protective immunity and allergic disease [135]. The primary mechanism of allergic diseases involves immunoglobulin E antibodies (IgE) that bind to an allergen and trigger the release of inflammatory chemicals [136].

Soybean is one of the foods that cause food-based allergic diseases in the U.S. and Europe [137]. Peanuts, tree nuts, cow milk, soybean, wheat, egg, fish, and crustaceans account for approximately 90% of food-based allergic diseases and are often called the “Big Eight” [138]. Soybean allergy affects approximately 0.4% of children, half as frequent as peanut allergy [139]. A population study with 133 patients with soybean allergy showed that 88% had concomitant peanut allergy [140]. To predict soybean allergy, soy-specific IgE levels in the blood are used as a biomarker, indicating that children with 20-30 kU/L of soy-specific IgE level will have a 50% chance of having soybean allergy [141]. Symptoms of a soybean allergy range from mild itching to systemic anaphylaxis and are comprised of four types: dermatological (hives, dermatitis, local swelling or eczema), gastrointestinal (abdominal pain, diarrhea, nausea or vomiting), respiratory (runny nose, breathing difficulty or asthma), and systemic (anaphylactic shock, organ failure, cardiac arrhythmia or death) [142].

Glycoproteins in soybean were found to be responsible allergens for IgE-related allergic disease, and there are eight allergens (Gly m 1-8) registered by the World Health Organization (WHO) named after soybean’s scientific name: *Glycine max* [137]. Various processing methods are utilized to reduce IgE-related allergens, including heat treatment, fermentation, enzymatic hydrolysis, or carbohydrate conjugation [143, 144].

## Concluding Remarks

Soybean and its bioactive components are significantly associated with reduced risks of many diseases, including cancer, T2DM, osteoporosis, and hot flashes, while showing marginal adverse effects (Fig. 2). The risks of breast, prostate, colorectal, ovarian, and lung cancer were inversely related to soybean consumption. Soybean consumption was also associated with reduced T2DM risk. Particularly, soy isoflavone consumption reduced bone mineral density loss of the femoral neck, lumbar spine, and hip showing preventive effects against osteoporosis. In randomized controlled trials, soy isoflavone reduced the frequency and severity of hot flashes in perimenopausal and postmenopausal women. Although genistein might negate the aromatase inhibitors for breast cancer treatment and approximately 0.4% of children are allergic to soy protein, making it half as frequent as peanut allergy, the benefits of soybean consumption far outweigh its adverse effects.

More randomized controlled trials of soybean using new molecular markers will further support its benefits, especially in cancer research. Soybean already showed its benefits against osteoporosis and hot flashes in meta-analyses of randomized controlled trials with large case numbers and defined groups [124, 128]. Although some soybean intervention studies with standard clinical trials in cancer were disappointing [145-147], some precision oncology-based approaches show promising correlations between soybean and genetic features [95, 96]. Soy intake was associated with a reduced risk of ER+/PR+ breast cancer in postmenopausal women and a reduced risk of ER-/PR- breast cancer in premenopausal women [96]. Soy food consumption was associated with a lower risk of recurrence in ER-negative and ER+/PR+ breast cancer patients [95]. Although patient recruitment challenges exist, randomized controlled trials of soybean-based on individuals’ genetic features and molecular markers will show the beneficial effects of soybean in reducing cancer incidence and recurrence. Given that the FDA planned to revoke the health claim of soy protein against coronary heart disease due to weak evidence from observational studies [19], randomized controlled trials of soybean in cancer will provide clear support.

Soybean is a healthy protein source compared to red meat, and considerable differences in daily soybean consumption in different countries will be remained as an essential factor. In the United States, for example, daily soy protein consumption is less than 1 g, and daily isoflavone intake is only 0.15-3 mg. Achieving a higher soy intake with soy protein-based products will be beneficial. However, there is a vast gap between soy intake in Asian countries where epidemiological evidence shows reduced disease risks. Supplements of soybean isoflavones or using soy protein as a food source will enhance the consumption of soybean bioactive components.

## Figures and Tables

**Table 1:** Chemical structures of soybean phytochemicals.